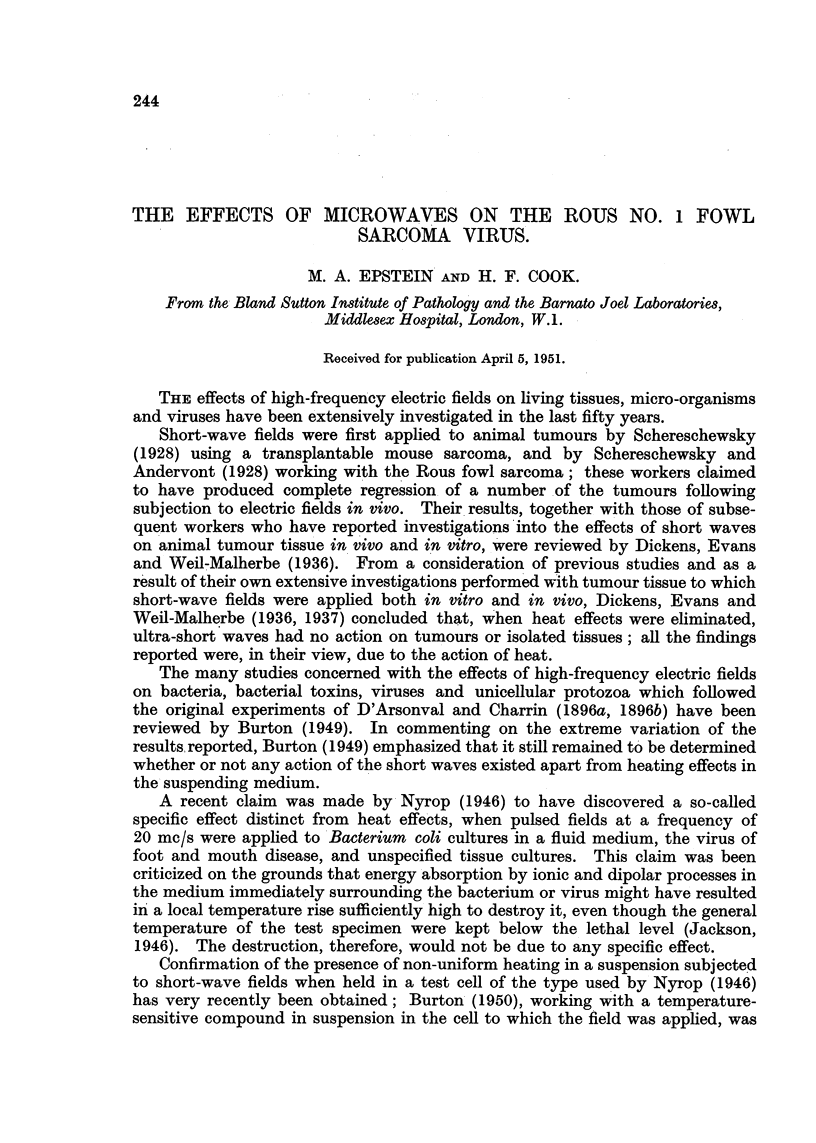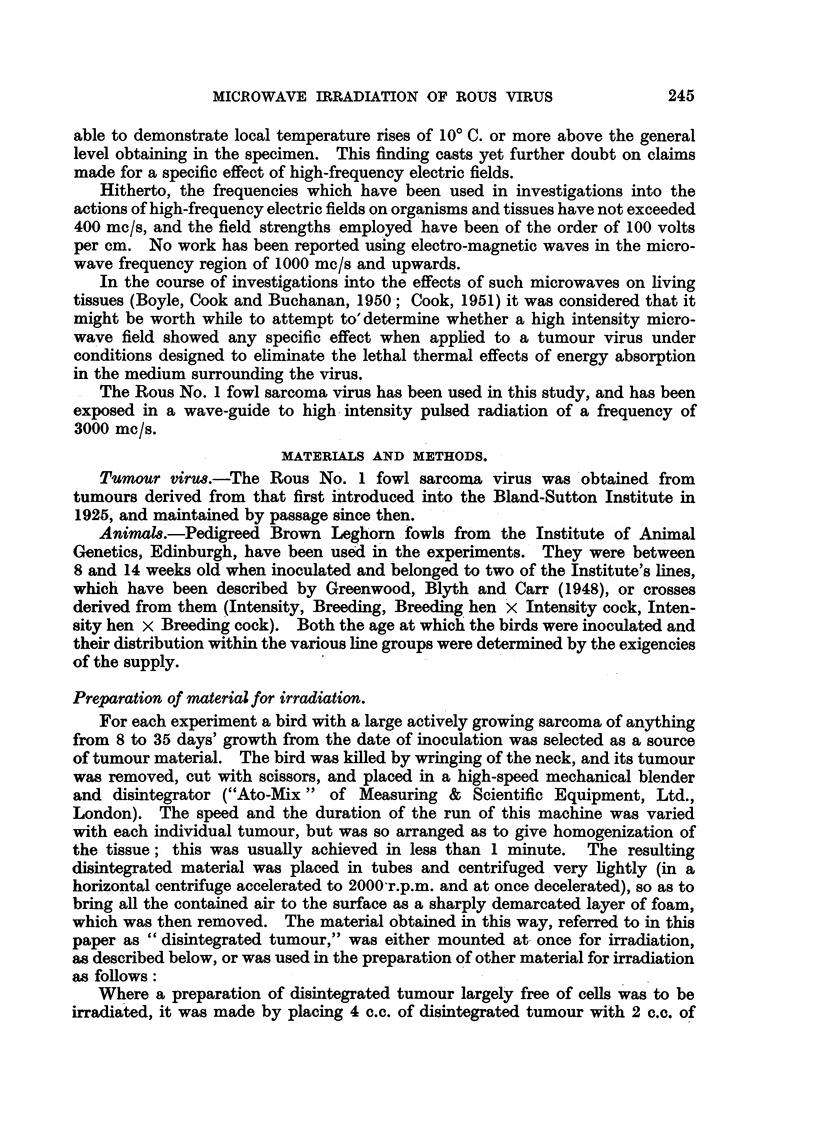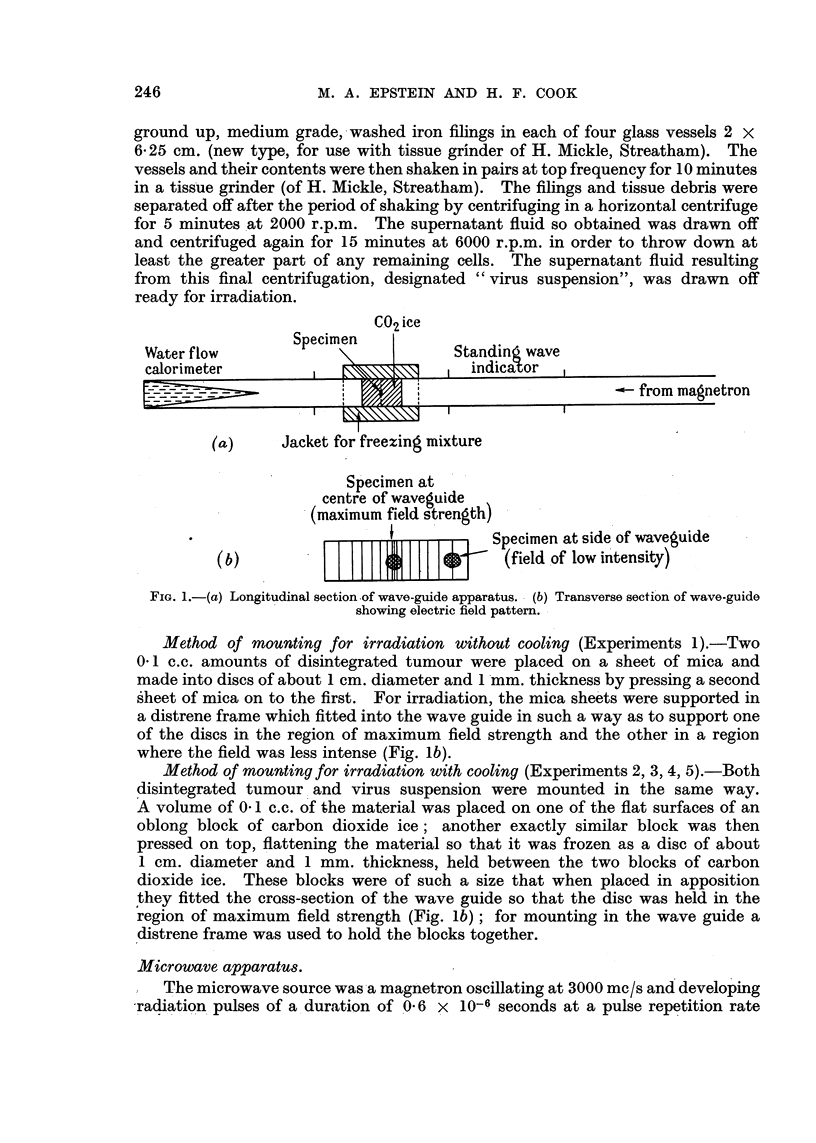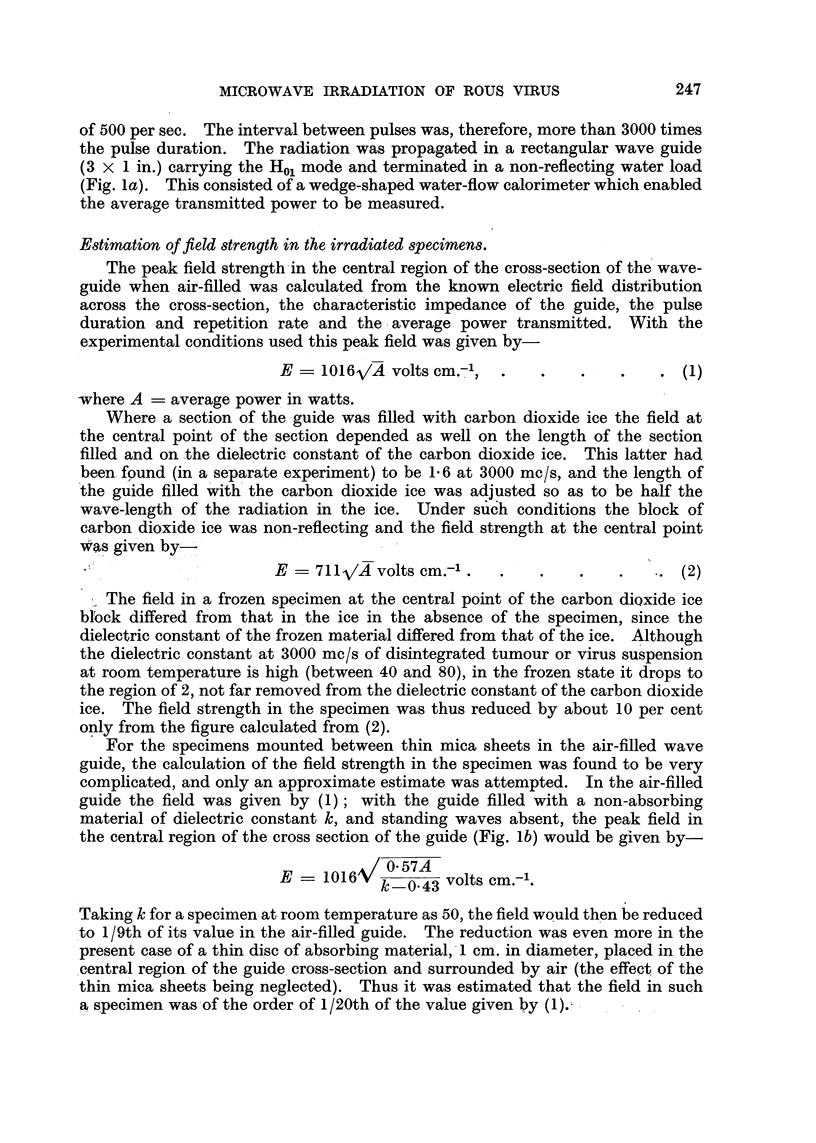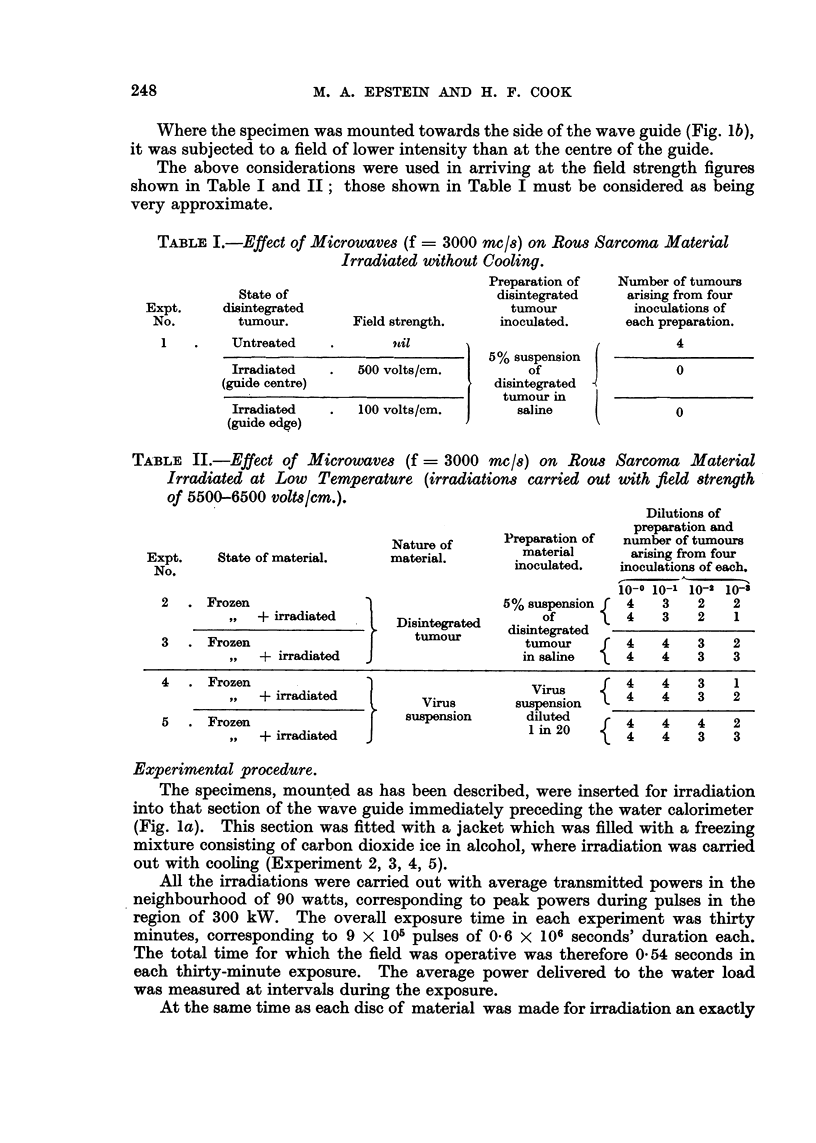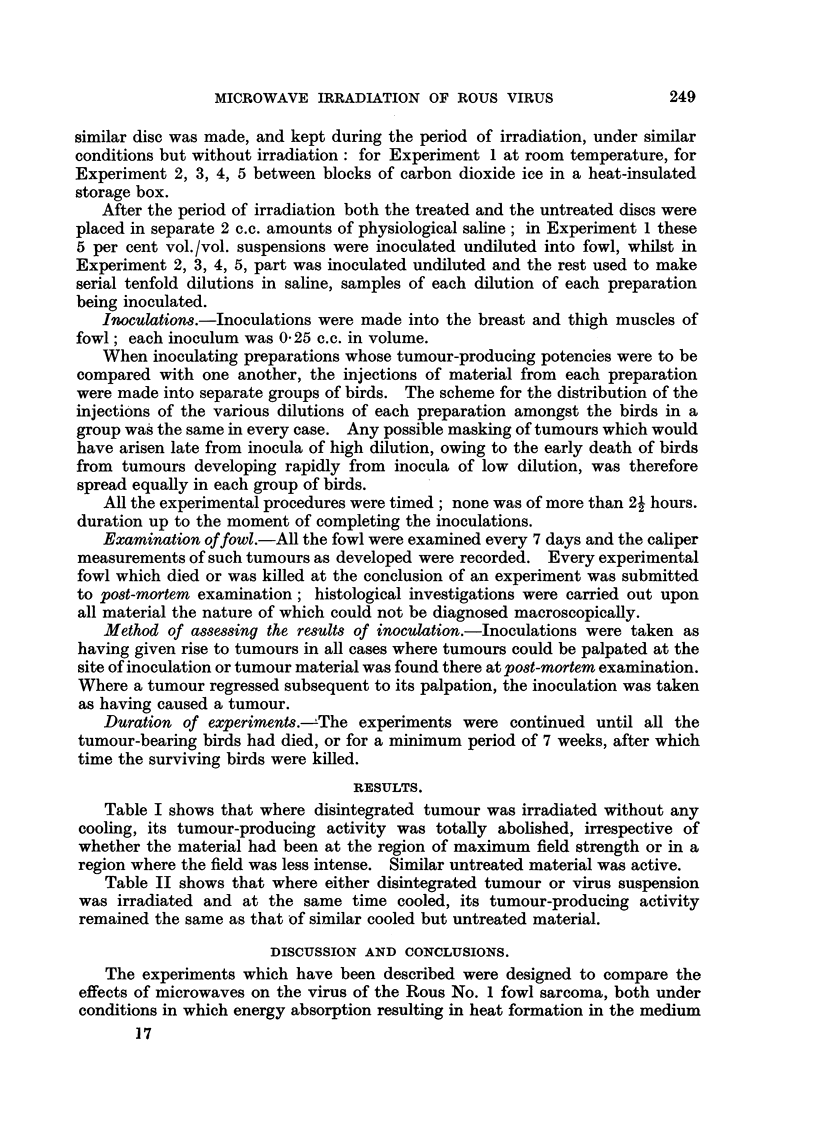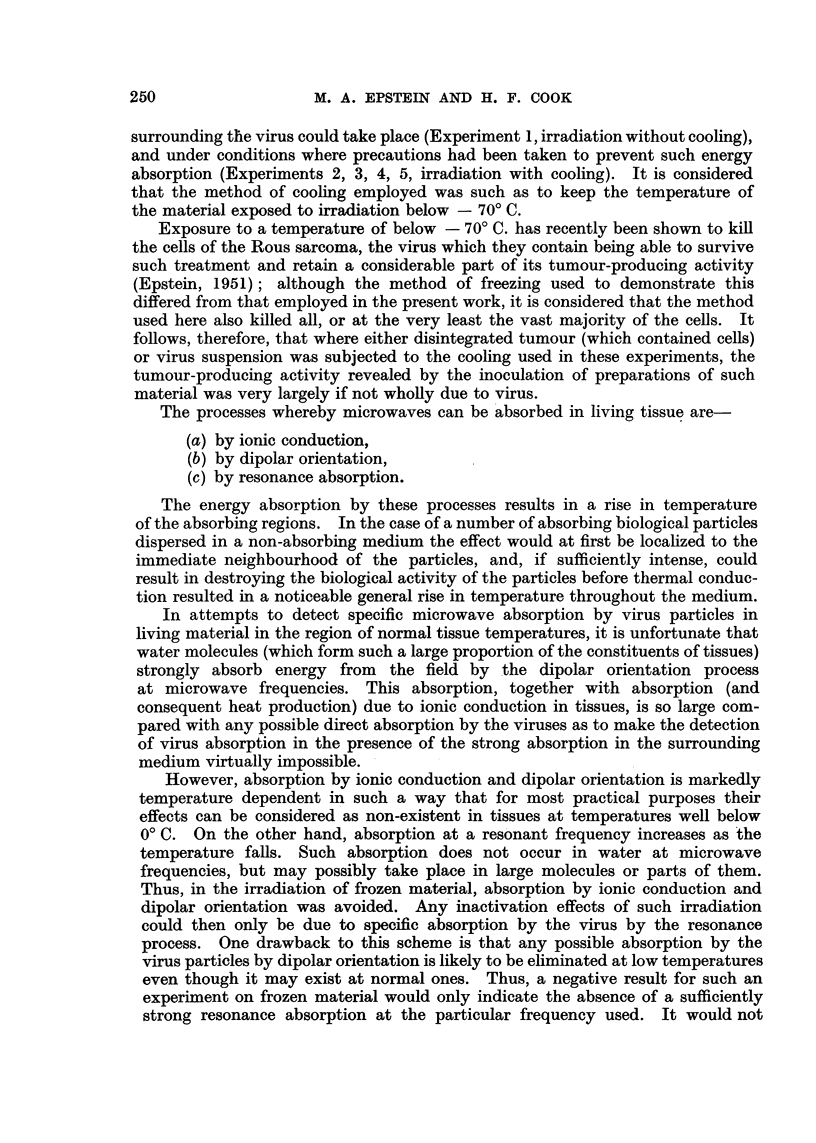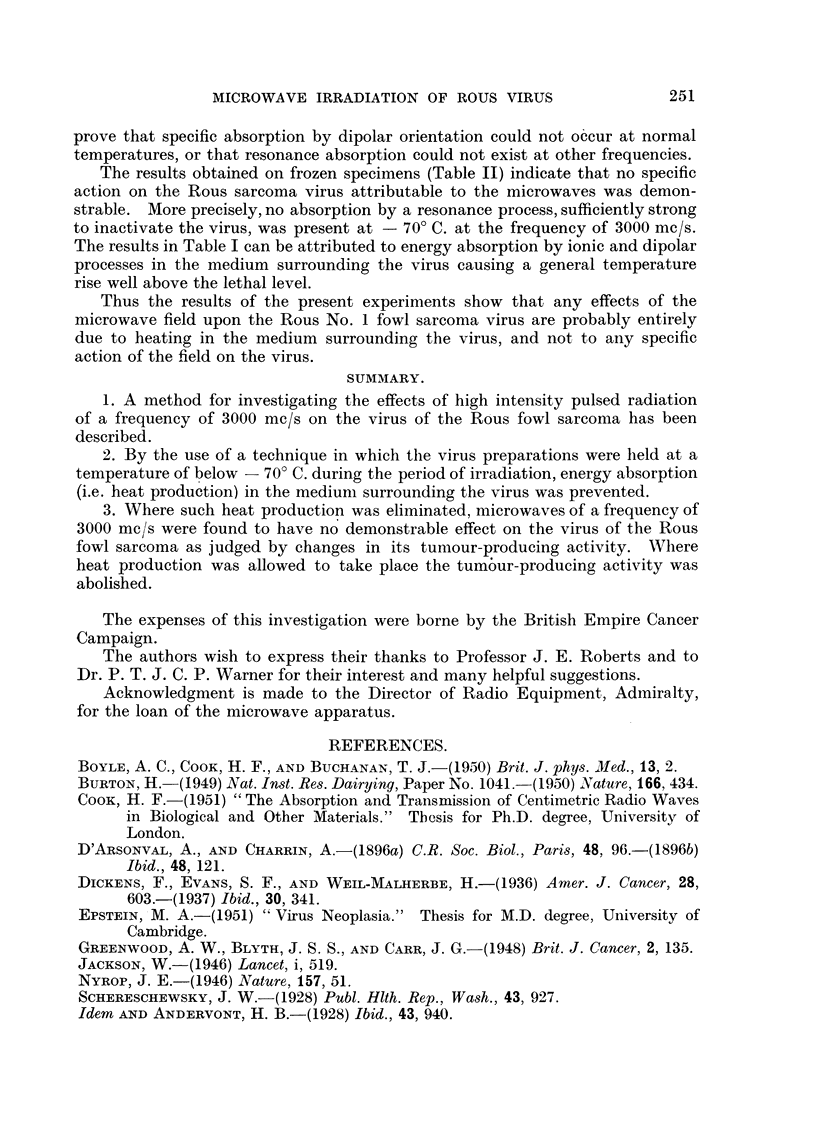# The Effects of Microwaves on the Rous No. 1 Fowl Sarcoma Virus

**DOI:** 10.1038/bjc.1951.25

**Published:** 1951-06

**Authors:** M. A. Epstein, H. F. Cook


### THE EFFECTS OF MICROWAVES ON THE ROUS NO. 1 FOWL SARCOMA VIRUS.

M. A. EPSTEIN AND H. F. COOK.

*From the Bland Sutton Institute of Pathology and the Barnato Joel Laboratories, Middlesex Hospital, London, W.1.*

Received for publication April 5, 1951.

THE effects of high-frequency electric fields on living tissues, micro-organisms and viruses have been extensively investigated in the last fifty years.

Short-wave fields were first applied to animal tumours by Schereschewsky (1928) using a transplantable mouse sarcoma, and by Schereschewsky and Andervont (1928) working with the Rous fowl sarcoma; these workers claimed to have produced complete regression of a number of the tumours following subjection to electric fields *in vivo*. Their results, together with those of subsequent workers who have reported investigations into the effects of short waves on animal tumour tissue *in vivo* and *in vitro*, were reviewed by Dickens, Evans and Weil-Malherbe (1936). From a consideration of previous studies and as a result of their own extensive investigations performed with tumour tissue to which short-wave fields were applied both *in vitro* and *in vivo*, Dickens, Evans and Weil-Malherbe (1936, 1937) concluded that, when heat effects were eliminated, ultra-short waves had no action on tumours or isolated tissues; all the findings reported were, in their view, due to the action of heat.

The many studies concerned with the effects of high-frequency electric fields on bacteria, bacterial toxins, viruses and unicellular protozoa which followed the original experiments of D'Arsonval and Charrin (1896*a*, 1896*b*) have been reviewed by Burton (1949). In commenting on the extreme variation of the results reported, Burton (1949) emphasized that it still remained to be determined whether or not any action of the short waves existed apart from heating effects in the suspending medium.

A recent claim was made by Nyrop (1946) to have discovered a so-called specific effect distinct from heat effects, when pulsed fields at a frequency of 20 mc/s were applied to *Bacterium coli* cultures in a fluid medium, the virus of foot and mouth disease, and unspecified tissue cultures. This claim was been criticized on the grounds that energy absorption by ionic and dipolar processes in the medium immediately surrounding the bacterium or virus might have resulted in a local temperature rise sufficiently high to destroy it, even though the general temperature of the test specimen were kept below the lethal level (Jackson, 1946). The destruction, therefore, would not be due to any specific effect.

Confirmation of the presence of non-uniform heating in a suspension subjected to short-wave fields when held in a test cell of the type used by Nyrop (1946) has very recently been obtained; Burton (1950), working with a temperature-sensitive compound in suspension in the cell to which the field was applied, was able to demonstrate local temperature rises of 10° C. or more above the general level obtaining in the specimen. This finding casts yet further doubt on claims made for a specific effect of high-frequency electric fields.

Hitherto, the frequencies which have been used in investigations into the actions of high-frequency electric fields on organisms and tissues have not exceeded 400 mc/s, and the field strengths employed have been of the order of 100 volts per cm. No work has been reported using electro-magnetic waves in the microwave frequency region of 1000 mc/s and upwards.

In the course of investigations into the effects of such microwaves on living tissues (Boyle, Cook and Buchanan, 1950; Cook, 1951) it was considered that it might be worth while to attempt to determine whether a high intensity microwave field showed any specific effect when applied to a tumour virus under conditions designed to eliminate the lethal thermal effects of energy absorption in the medium surrounding the virus.

The Rous No. 1 fowl sarcoma virus has been used in this study, and has been exposed in a wave-guide to high intensity pulsed radiation of a frequency of 3000 mc/s.

### MATERIALS AND METHODS.

*Tumour virus.*—The Rous No. 1 fowl sarcoma virus was obtained from tumours derived from that first introduced into the Bland-Sutton Institute in 1925, and maintained by passage since then.

*Animals.*—Pedigreed Brown Leghorn fowls from the Institute of Animal Genetics, Edinburgh, have been used in the experiments. They were between 8 and 14 weeks old when inoculated and belonged to two of the Institute's lines, which have been described by Greenwood, Blyth and Carr (1948), or crosses derived from them (Intensity, Breeding, Breeding hen × Intensity cock, Intensity hen × Breeding cock). Both the age at which the birds were inoculated and their distribution within the various line groups were determined by the exigencies of the supply.

### *Preparation of material for irradiation.*

For each experiment a bird with a large actively growing sarcoma of anything from 8 to 35 days' growth from the date of inoculation was selected as a source of tumour material. The bird was killed by wringing of the neck, and its tumour was removed, cut with scissors, and placed in a high-speed mechanical blender and disintegrator ("Ato-Mix" of Measuring & Scientific Equipment, Ltd., London). The speed and the duration of the run of this machine was varied with each individual tumour, but was so arranged as to give homogenization of the tissue; this was usually achieved in less than 1 minute. The resulting disintegrated material was placed in tubes and centrifuged very lightly (in a horizontal centrifuge accelerated to 2000 r.p.m. and at once decelerated), so as to bring all the contained air to the surface as a sharply demarcated layer of foam, which was then removed. The material obtained in this way, referred to in this paper as "disintegrated tumour," was either mounted at once for irradiation, as described below, or was used in the preparation of other material for irradiation as follows:

Where a preparation of disintegrated tumour largely free of cells was to be irradiated, it was made by placing 4 c.c. of disintegrated tumour with 2 c.c. of ground up, medium grade, washed iron filings in each of four glass vessels 2 × 6·25 cm. (new type, for use with tissue grinder of H. Mickle, Streatham). The vessels and their contents were then shaken in pairs at top frequency for 10 minutes in a tissue grinder (of H. Mickle, Streatham). The filings and tissue debris were separated off after the period of shaking by centrifuging in a horizontal centrifuge for 5 minutes at 2000 r.p.m. The supernatant fluid so obtained was drawn off and centrifuged again for 15 minutes at 6000 r.p.m. in order to throw down at least the greater part of any remaining cells. The supernatant fluid resulting from this final centrifugation, designated "virus suspension", was drawn off ready for irradiation.

FIG. 1.—(*a*) Longitudinal section of wave-guide apparatus. (*b*) Transverse section of wave-guide showing electric field pattern.

*Method of mounting for irradiation without cooling* (Experiments 1).—Two 0·1 c.c. amounts of disintegrated tumour were placed on a sheet of mica and made into discs of about 1 cm. diameter and 1 mm. thickness by pressing a second sheet of mica on to the first. For irradiation, the mica sheets were supported in a distrene frame which fitted into the wave guide in such a way as to support one of the discs in the region of maximum field strength and the other in a region where the field was less intense (Fig. 1*b*).

*Method of mounting for irradiation with cooling* (Experiments 2, 3, 4, 5).—Both disintegrated tumour and virus suspension were mounted in the same way. A volume of 0·1 c.c. of the material was placed on one of the flat surfaces of an oblong block of carbon dioxide ice; another exactly similar block was then pressed on top, flattening the material so that it was frozen as a disc of about 1 cm. diameter and 1 mm. thickness, held between the two blocks of carbon dioxide ice. These blocks were of such a size that when placed in apposition they fitted the cross-section of the wave guide so that the disc was held in the region of maximum field strength (Fig. 1*b*); for mounting in the wave guide a distrene frame was used to hold the blocks together.

*Microwave apparatus.*

The microwave source was a magnetron oscillating at 3000 mc/s and developing radiation pulses of a duration of 0·6 × $10^{-6}$ seconds at a pulse repetition rate of 500 per sec. The interval between pulses was, therefore, more than 3000 times the pulse duration. The radiation was propagated in a rectangular wave guide ($3 \times 1$ in.) carrying the $H_{01}$ mode and terminated in a non-reflecting water load (Fig. 1*a*). This consisted of a wedge-shaped water-flow calorimeter which enabled the average transmitted power to be measured.

*Estimation of field strength in the irradiated specimens.*

The peak field strength in the central region of the cross-section of the wave-guide when air-filled was calculated from the known electric field distribution across the cross-section, the characteristic impedance of the guide, the pulse duration and repetition rate and the average power transmitted. With the experimental conditions used this peak field was given by—

$$E = 1016\sqrt{A} \text{ volts cm.}^{-1}, \quad . \quad . \quad . \quad . \quad . \quad (1)$$

where $A$ = average power in watts.

Where a section of the guide was filled with carbon dioxide ice the field at the central point of the section depended as well on the length of the section filled and on the dielectric constant of the carbon dioxide ice. This latter had been found (in a separate experiment) to be 1·6 at 3000 mc/s, and the length of the guide filled with the carbon dioxide ice was adjusted so as to be half the wave-length of the radiation in the ice. Under such conditions the block of carbon dioxide ice was non-reflecting and the field strength at the central point was given by—

$$E = 711\sqrt{A} \text{ volts cm.}^{-1} \quad . \quad . \quad . \quad . \quad . \quad . \quad (2)$$

The field in a frozen specimen at the central point of the carbon dioxide ice block differed from that in the ice in the absence of the specimen, since the dielectric constant of the frozen material differed from that of the ice. Although the dielectric constant at 3000 mc/s of disintegrated tumour or virus suspension at room temperature is high (between 40 and 80), in the frozen state it drops to the region of 2, not far removed from the dielectric constant of the carbon dioxide ice. The field strength in the specimen was thus reduced by about 10 per cent only from the figure calculated from (2).

For the specimens mounted between thin mica sheets in the air-filled wave guide, the calculation of the field strength in the specimen was found to be very complicated, and only an approximate estimate was attempted. In the air-filled guide the field was given by (1); with the guide filled with a non-absorbing material of dielectric constant $k$, and standing waves absent, the peak field in the central region of the cross section of the guide (Fig. 1*b*) would be given by—

$$E = 1016\sqrt{\frac{0\cdot57A}{k-0\cdot43}} \text{ volts cm.}^{-1}.$$

Taking $k$ for a specimen at room temperature as 50, the field would then be reduced to 1/9th of its value in the air-filled guide. The reduction was even more in the present case of a thin disc of absorbing material, 1 cm. in diameter, placed in the central region of the guide cross-section and surrounded by air (the effect of the thin mica sheets being neglected). Thus it was estimated that the field in such a specimen was of the order of 1/20th of the value given by (1).

Where the specimen was mounted towards the side of the wave guide (Fig. 1*b*), it was subjected to a field of lower intensity than at the centre of the guide.

The above considerations were used in arriving at the field strength figures shown in Table I and II; those shown in Table I must be considered as being very approximate.

TABLE I.—*Effect of Microwaves* (f = 3000 *mc/s*) *on Rous Sarcoma Material Irradiated without Cooling.*

| Expt. No. | State of disintegrated tumour. | Field strength. | Preparation of disintegrated tumour inoculated. | Number of tumours arising from four inoculations of each preparation. |
|---|---|---|---|---|
| 1 | Untreated | *nil* | 5% suspension of disintegrated tumour in saline | 4 |
| | Irradiated (guide centre) | 500 volts/cm. | | 0 |
| | Irradiated (guide edge) | 100 volts/cm. | | 0 |

TABLE II.—*Effect of Microwaves* (f = 3000 *mc/s*) *on Rous Sarcoma Material Irradiated at Low Temperature* (*irradiations carried out with field strength of* 5500–6500 *volts/cm.*).

| Expt. No. | State of material. | Nature of material. | Preparation of material inoculated. | Dilutions of preparation and number of tumours arising from four inoculations of each. $10^{-0}$ | $10^{-1}$ | $10^{-2}$ | $10^{-3}$ |
|---|---|---|---|---|---|---|---|
| 2 | Frozen | Disintegrated tumour | 5% suspension of disintegrated tumour in saline | 4 | 3 | 2 | 2 |
| | „ + irradiated | | | 4 | 3 | 2 | 1 |
| 3 | Frozen | | | 4 | 4 | 3 | 2 |
| | „ + irradiated | | | 4 | 4 | 3 | 3 |
| 4 | Frozen | Virus suspension | Virus suspension diluted 1 in 20 | 4 | 4 | 3 | 1 |
| | „ + irradiated | | | 4 | 4 | 3 | 2 |
| 5 | Frozen | | | 4 | 4 | 4 | 2 |
| | „ + irradiated | | | 4 | 4 | 3 | 3 |

*Experimental procedure.*

The specimens, mounted as has been described, were inserted for irradiation into that section of the wave guide immediately preceding the water calorimeter (Fig. 1*a*). This section was fitted with a jacket which was filled with a freezing mixture consisting of carbon dioxide ice in alcohol, where irradiation was carried out with cooling (Experiment 2, 3, 4, 5).

All the irradiations were carried out with average transmitted powers in the neighbourhood of 90 watts, corresponding to peak powers during pulses in the region of 300 kW. The overall exposure time in each experiment was thirty minutes, corresponding to $9 \times 10^5$ pulses of $0{\cdot}6 \times 10^6$ seconds' duration each. The total time for which the field was operative was therefore 0·54 seconds in each thirty-minute exposure. The average power delivered to the water load was measured at intervals during the exposure.

At the same time as each disc of material was made for irradiation an exactly similar disc was made, and kept during the period of irradiation, under similar conditions but without irradiation : for Experiment 1 at room temperature, for Experiment 2, 3, 4, 5 between blocks of carbon dioxide ice in a heat-insulated storage box.

After the period of irradiation both the treated and the untreated discs were placed in separate 2 c.c. amounts of physiological saline ; in Experiment 1 these 5 per cent vol./vol. suspensions were inoculated undiluted into fowl, whilst in Experiment 2, 3, 4, 5, part was inoculated undiluted and the rest used to make serial tenfold dilutions in saline, samples of each dilution of each preparation being inoculated.

*Inoculations.*—Inoculations were made into the breast and thigh muscles of fowl ; each inoculum was 0·25 c.c. in volume.

When inoculating preparations whose tumour-producing potencies were to be compared with one another, the injections of material from each preparation were made into separate groups of birds. The scheme for the distribution of the injections of the various dilutions of each preparation amongst the birds in a group was the same in every case. Any possible masking of tumours which would have arisen late from inocula of high dilution, owing to the early death of birds from tumours developing rapidly from inocula of low dilution, was therefore spread equally in each group of birds.

All the experimental procedures were timed ; none was of more than 2½ hours. duration up to the moment of completing the inoculations.

*Examination of fowl.*—All the fowl were examined every 7 days and the caliper measurements of such tumours as developed were recorded. Every experimental fowl which died or was killed at the conclusion of an experiment was submitted to *post-mortem* examination ; histological investigations were carried out upon all material the nature of which could not be diagnosed macroscopically.

*Method of assessing the results of inoculation.*—Inoculations were taken as having given rise to tumours in all cases where tumours could be palpated at the site of inoculation or tumour material was found there at *post-mortem* examination. Where a tumour regressed subsequent to its palpation, the inoculation was taken as having caused a tumour.

*Duration of experiments.*—The experiments were continued until all the tumour-bearing birds had died, or for a minimum period of 7 weeks, after which time the surviving birds were killed.

### RESULTS.

Table I shows that where disintegrated tumour was irradiated without any cooling, its tumour-producing activity was totally abolished, irrespective of whether the material had been at the region of maximum field strength or in a region where the field was less intense. Similar untreated material was active.

Table II shows that where either disintegrated tumour or virus suspension was irradiated and at the same time cooled, its tumour-producing activity remained the same as that of similar cooled but untreated material.

### DISCUSSION AND CONCLUSIONS.

The experiments which have been described were designed to compare the effects of microwaves on the virus of the Rous No. 1 fowl sarcoma, both under conditions in which energy absorption resulting in heat formation in the medium surrounding the virus could take place (Experiment 1, irradiation without cooling), and under conditions where precautions had been taken to prevent such energy absorption (Experiments 2, 3, 4, 5, irradiation with cooling). It is considered that the method of cooling employed was such as to keep the temperature of the material exposed to irradiation below — 70° C.

Exposure to a temperature of below — 70° C. has recently been shown to kill the cells of the Rous sarcoma, the virus which they contain being able to survive such treatment and retain a considerable part of its tumour-producing activity (Epstein, 1951); although the method of freezing used to demonstrate this differed from that employed in the present work, it is considered that the method used here also killed all, or at the very least the vast majority of the cells. It follows, therefore, that where either disintegrated tumour (which contained cells) or virus suspension was subjected to the cooling used in these experiments, the tumour-producing activity revealed by the inoculation of preparations of such material was very largely if not wholly due to virus.

The processes whereby microwaves can be absorbed in living tissue are—

(*a*) by ionic conduction,
(*b*) by dipolar orientation,
(*c*) by resonance absorption.

The energy absorption by these processes results in a rise in temperature of the absorbing regions. In the case of a number of absorbing biological particles dispersed in a non-absorbing medium the effect would at first be localized to the immediate neighbourhood of the particles, and, if sufficiently intense, could result in destroying the biological activity of the particles before thermal conduction resulted in a noticeable general rise in temperature throughout the medium.

In attempts to detect specific microwave absorption by virus particles in living material in the region of normal tissue temperatures, it is unfortunate that water molecules (which form such a large proportion of the constituents of tissues) strongly absorb energy from the field by the dipolar orientation process at microwave frequencies. This absorption, together with absorption (and consequent heat production) due to ionic conduction in tissues, is so large compared with any possible direct absorption by the viruses as to make the detection of virus absorption in the presence of the strong absorption in the surrounding medium virtually impossible.

However, absorption by ionic conduction and dipolar orientation is markedly temperature dependent in such a way that for most practical purposes their effects can be considered as non-existent in tissues at temperatures well below 0° C. On the other hand, absorption at a resonant frequency increases as the temperature falls. Such absorption does not occur in water at microwave frequencies, but may possibly take place in large molecules or parts of them. Thus, in the irradiation of frozen material, absorption by ionic conduction and dipolar orientation was avoided. Any inactivation effects of such irradiation could then only be due to specific absorption by the virus by the resonance process. One drawback to this scheme is that any possible absorption by the virus particles by dipolar orientation is likely to be eliminated at low temperatures even though it may exist at normal ones. Thus, a negative result for such an experiment on frozen material would only indicate the absence of a sufficiently strong resonance absorption at the particular frequency used. It would not prove that specific absorption by dipolar orientation could not occur at normal temperatures, or that resonance absorption could not exist at other frequencies.

The results obtained on frozen specimens (Table II) indicate that no specific action on the Rous sarcoma virus attributable to the microwaves was demonstrable. More precisely, no absorption by a resonance process, sufficiently strong to inactivate the virus, was present at $-70°$ C. at the frequency of 3000 mc/s. The results in Table I can be attributed to energy absorption by ionic and dipolar processes in the medium surrounding the virus causing a general temperature rise well above the lethal level.

Thus the results of the present experiments show that any effects of the microwave field upon the Rous No. 1 fowl sarcoma virus are probably entirely due to heating in the medium surrounding the virus, and not to any specific action of the field on the virus.

SUMMARY.

1. A method for investigating the effects of high intensity pulsed radiation of a frequency of 3000 mc/s on the virus of the Rous fowl sarcoma has been described.

2. By the use of a technique in which the virus preparations were held at a temperature of below $-70°$ C. during the period of irradiation, energy absorption (i.e. heat production) in the medium surrounding the virus was prevented.

3. Where such heat production was eliminated, microwaves of a frequency of 3000 mc/s were found to have no demonstrable effect on the virus of the Rous fowl sarcoma as judged by changes in its tumour-producing activity. Where heat production was allowed to take place the tumour-producing activity was abolished.

The expenses of this investigation were borne by the British Empire Cancer Campaign.

The authors wish to express their thanks to Professor J. E. Roberts and to Dr. P. T. J. C. P. Warner for their interest and many helpful suggestions.

Acknowledgment is made to the Director of Radio Equipment, Admiralty, for the loan of the microwave apparatus.

### REFERENCES.

BOYLE, A. C., COOK, H. F., AND BUCHANAN, T. J.—(1950) *Brit. J. phys. Med.*, **13**, 2.

BURTON, H.—(1949) *Nat. Inst. Res. Dairying*, Paper No. 1041.—(1950) *Nature*, **166**, 434.

COOK, H. F.—(1951) "The Absorption and Transmission of Centimetric Radio Waves in Biological and Other Materials." Thesis for Ph.D. degree, University of London.

D'ARSONVAL, A., AND CHARRIN, A.—(1896*a*) *C.R. Soc. Biol., Paris*, **48**, 96.—(1896*b*) *Ibid.*, **48**, 121.

DICKENS, F., EVANS, S. F., AND WEIL-MALHERBE, H.—(1936) *Amer. J. Cancer*, **28**, 603.—(1937) *Ibid.*, **30**, 341.

EPSTEIN, M. A.—(1951) "Virus Neoplasia." Thesis for M.D. degree, University of Cambridge.

GREENWOOD, A. W., BLYTH, J. S. S., AND CARR, J. G.—(1948) *Brit. J. Cancer*, **2**, 135.

JACKSON, W.—(1946) *Lancet*, i, 519.

NYROP, J. E.—(1946) *Nature*, **157**, 51.

SCHERESCHEWSKY, J. W.—(1928) *Publ. Hlth. Rep., Wash.*, **43**, 927.

*Idem* AND ANDERVONT, H. B.—(1928) *Ibid.*, **43**, 940.